# Extraverted Children Are More Biased by Bowl Sizes than Introverts

**DOI:** 10.1371/journal.pone.0078224

**Published:** 2013-10-30

**Authors:** Koert van Ittersum, Brian Wansink

**Affiliations:** 1 Department of Marketing, University of Groningen, Groningen, The Netherlands; 2 Charles H. Dyson School of Applied Economics and Management, Cornell University, Ithaca, New York, United States of America; University of Granada, Spain

## Abstract

Extraverted children are hypothesized to be most at risk for over-serving and overeating due to environmental cues – such as the size of dinnerware. A within-subject field study of elementary school students found that extraverted children served themselves 33.1% more cereal in larger bowls (16-oz) than in smaller (12-oz) bowls, whereas introverted children were unaffected by bowl size (+5.6%, ns). However, when children were asked by adults how much cereal they wanted to eat, both extraverted and introverted children requested more cereal when given a large versus small bowl. Insofar as extraverted children appear to be more biased by environmental cues, this pilot study suggests different serving styles are recommended for parents and other caregivers. They should serve extraverts, but allow introverts to serve themselves. Still, since the average child still served 23.2% more when serving themselves than when served by an adult, it might be best for caregivers to do the serving whenever possible – especially for extraverted children.

## Introduction

As the size of dinnerware increases, so do self-servings and consumption of food by adults [1], [2]. Yet, while this happens with adults, it is not known whether the same visual bias influences children and if it influences some more extremely than others [3], [4]. With increasing concerns about childhood obesity, knowing what types of children are most at risk of being influenced by such external cues could sharpen the focus of caregiver interventions [5], [6].

One characteristic that has shown to be correlated with overconsumption and obesity in adults is extraversion [7]–[11]. Behavioral differences between extraverted and introverted individuals have been attributed to differences in the self-regulation of arousal [12]–[14]. Introverts typically show higher levels of cortical arousal than extraverts, as a result of which introverts seek a reduction of their arousal levels [14], whereas extraverts may additionally increase their arousal through food consumption [15].

There is supporting evidence that extraverted children might also be more influenced by these external cues than introverts. First, extraverted children tend to exhibit lower self-consciousness [16] and have an external processing style, while introverted children have an internal processing style that involves reflection and consideration [17]. Furthermore, extraverts exhibit faster movement times than introverts (possibly because of differences in motor processes) [18]. This may suggest that introverts are more cautious in their actions, which reduces the self-serving of food. Since introverts are more self-conscious, have internal processing styles that make them less susceptible to environmental cues, are more likely to rely on internal standards such as food portions, and are more cautious in their behavioral actions, we hypothesize that when they serve themselves, the serving size of introverted children is less influenced by dinnerware size than it is for extraverted children.

However, a different dynamic might occur when parents or caregivers are serving food to children – which often happens for children under the age of 12 [19]. Although introverted children may have more internal control when serving their own food, this may change in the presence of others [20], [21]. For instance, when a second person becomes more involved, task performance of introverts has been shown to reduce significantly [22]. This is thought to be because the presence of this second person may lead the introvert to give up or relinquish their control of the situation [15]. Furthermore, introversion is strongly associated with behavioral and mental disengagement – that is, they are more likely to give up on the attempt to attain the goal with which a stressor is interfering [23]. In the context of serving food, we propose that when food is being served by a parent or adult, the difference in the amount of food requested by introverted and extraverted children becomes less dramatic [24].

## Methods

To examine the impact of extraversion on serving behavior, we conducted a within-subject study involving 18 elementary school children (66.7% female). This study was approved by the Cornell University Institutional Review Board, and parents or guardians provided written consent for their children to participate in this study. The average age of the children was 8.3 (age range 6–12). The study took place during four subsequent breakfasts. During two of the days, a server affiliated with the school cafeteria served the children in either a larger (16-oz) versus a smaller (12-oz) bowl. Children were asked to indicate how much cereal and milk they wanted. During the other two days children served themselves. Hidden scales embedded in the table with remote sensors measured how much cereal and milk each child served themselves or was served. How much they consumed was determined by subtracting the amount of cereal and milk that remained at the end of breakfast.

To measure the extraversion of each child, four attending teachers and counselors were simply asked to rate each child on the degree to which they were “extraverted” and again on the degree to which they were “introverted” (1 = strongly disagree; 9 = strongly agree). The names of the children were listed in two different orders to reduce the risk of order bias. One counselor and one teacher rated each child’s extraversion in a list that was presented in alphabetical order and the second counselor and the second teacher rated them in a list that was presented in reverse alphabetical order. After reverse scaling the introversion scores, the average of the extraversion and introversion scores was calculated across the four teachers and counselors (Cronbach’s α = .87). The average of these scores was used to classify the children as either a relative “extravert” or a relative “introvert” based on a median-split.

To examine the external validity of both scores, we conducted a series of correlation analyses (involving other personality traits collected during the study) and consistent with literature (e.g., [20], [25]) find that the extraversion positively correlated with outgoing (*r* = .88, *p*<.01), adventurous (*r* = .69, *p*<.01), energetic (*r* = .77, *p*<.01), impulsive (*r* = .67, *p*<.01), and negatively correlated with obedient (*r* = −.33, *p*<.10), cautious (*r* = −.57, *p*<.01), and concentrated (*r* = −.33, *p*<.10). We also examined the hypotheses based on the averages using OLS regression analyses. As our conclusions do not change, we present the dummy-variable results for readability purposes. Examining this variable as binary aids in the reporting of results and the ability to clearly illustrate them in a figure. A power analysis indicated a power of 0.92 for detecting an effect size of 0.50 at the 5% confidence level with a sample of 15.

## Results

Analysis of variance revealed that relatively extraverted children served and consumed 28.9% more grams of cereal than their introverted classmates (268.49 vs. 208.29; *F*(1,14) = 4.88, *p* = .04). Additionally, when all children served themselves, they served and consumed 23.2% more than when an adult served them (263.18 vs. 213.60; *F*(1,14) = 3.40, *p* = .09).

A significant three-way interaction between bowl size, extraversion, and server (*F*(1,14) = 5.76, *p* = .02) tentatively suggests that the effect of bowl size on serving size may be moderated by extraversion and the server. First, consistent with expectations, introverted children were less sensitive to the size of the bowl than extraverted children when they serve themselves (*F*(1, 14) = 4.65, *p* = .05). While the introverted children served an insignificant 5.6% more in the larger than the smaller bowl (216.59 vs. 228.67; *t*(8) = .67, *p*>.10), the extraverted children served a significant 33.1% more in the larger than the smaller bowl (260.65 vs. 346.82; *t*(8) = 2.55, *p* = .03).

**Figure 1:**
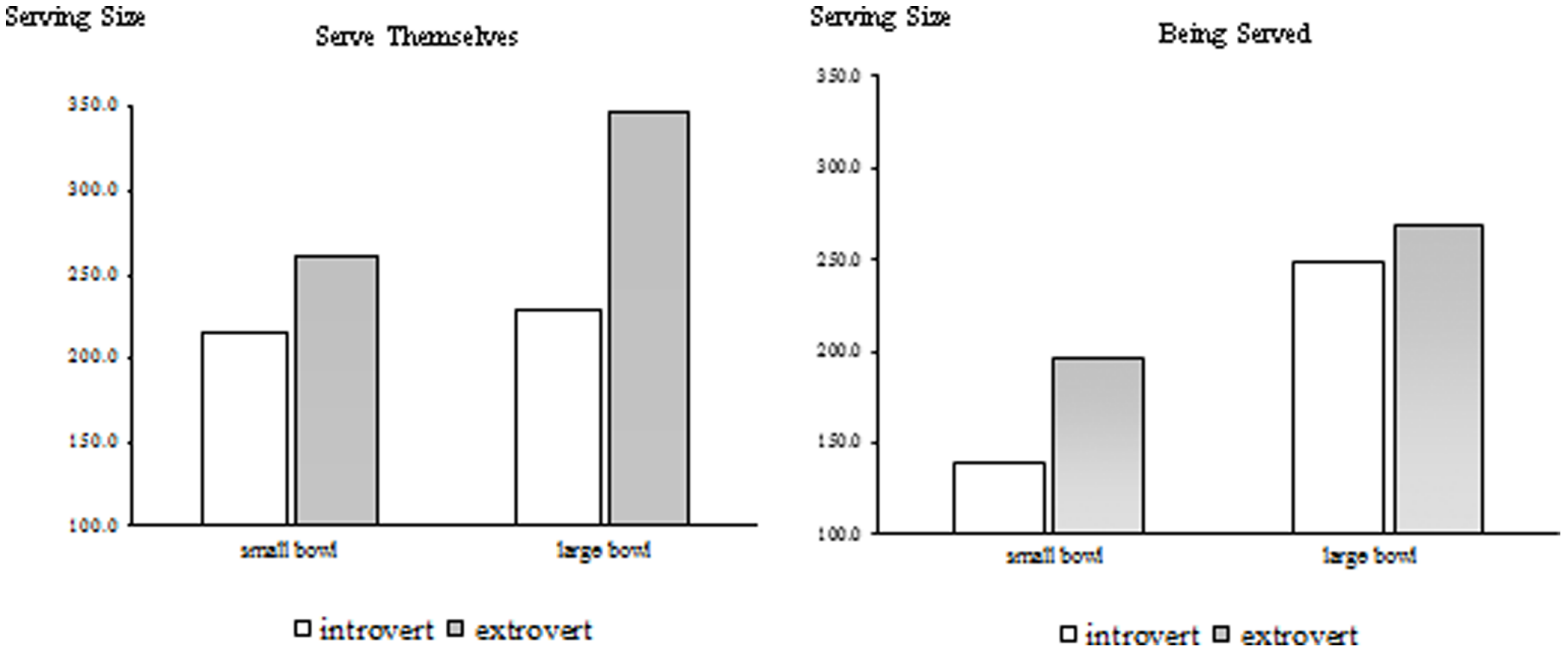
Extraverted Children Tend to Overserve Themselves.

When an adult became involved, however, these biases changed, as indicated in Figure 1. When an adult served the cereal and milk, introverted children became more sensitive to the size of the bowl than extraverted children. Although the interaction was not significant (*F*(1, 14) = 1.14, *p* = .30), introverted children ended up serving 79.5% more in the larger versus the smaller bowl (138.76 vs. 249.13; *t*(8) = 2.85, *p* = .02), which is substantially more than the 36.1% extra that extraverted children served in their large versus the small bowls (197.61 vs. 268.88; t(8) = 1.65, *p* = .14).

## General Discussion

Extraversion may be a critical personality characteristic when examining the susceptibility to environmental cues. When introverted children serve themselves they may be less influenced by environmental cues – such as bowl size – however this resolve disappears when served by a caregiver. In contrast, extraverted children seemingly benefit from having someone else serve their food for them.

Conducting this in a field study setting where children are eating their daily breakfast increases confidence that this relationship between extraversion and food intake is both relevant and robust [15]. While many personality studies have relatively large numbers of subjects for reliability, a pilot study showed the effect size for this intervention merited a smaller number of children who were repeatedly examined. Future research could examine whether this same impact happened with a more diverse population of children. In addition, there are a wide range of other factors that might influence the extent to which an extraverted person consumes more than an introvert. The mechanisms driving this may vary based on the anxiety of extraverts or the self-consciousness of introverts. One extension would be to observe if extraverts and introverts behave more similarly when eating alone.

As the size of bowls increases and a child serves themselves, extraverted children are most at risk to overserve themselves. When a child is served by another – such as a parent or caregiver – and asked how much they wish to eat, both introverted and extraverted children request over 50% more if the caregiver uses a larger bowl than a smaller one. Insofar as extraverted children appear to be more at risk for influence by environmental cues, there are two different serving recommendations for parents. Extraverted children should be served by an adult, and introverted children should be allowed to serve themselves. Still, since the average child served 23.2% more when serving themselves than when served by an adult, it might be best for caregivers to do the serving whenever possible – but especially for extraverted children.
